# Effect of home ultrasound in patients with previous late pregnancy loss: an interventional study

**DOI:** 10.1007/s00404-026-08429-9

**Published:** 2026-04-15

**Authors:** Liat Mor, Hagit Eisenberg, Liliya Tamayev, Daniel Tairy, Ben Oren, Yael Ganor Paz, Michal Levy, Eran Weiner, Giulia Barda

**Affiliations:** 1 Department of Obstetrics and Gynecology, The Edith Wolfson Medical Center, P.O. Box 5, 58100 Holon, Israel; 2 Affiliated with the Faculty of Medicine, Tel Aviv University, Tel Aviv, Israel

**Keywords:** Stillbirth, Pregnancy loss, Telemedicine, Anxiety, Attachment

## Abstract

**Purpose:**

To evaluate the effect of incorporating twice-weekly telemedicine home ultrasound sessions on maternal anxiety and antenatal attachment in pregnant patients with a history of late pregnancy loss.

**Methods:**

In this quasi-randomized trial, pregnant patients with a previous pregnancy loss beyond 20 weeks of gestation were randomized per day of enrollment to standard high-risk care (control) or additional twice-weekly home ultrasound sessions (intervention). Maternal anxiety and antenatal attachment were assessed at baseline, mid-pregnancy, and final prenatal visit using the State–Trait Anxiety Inventory Scale (STAI-S) and the Maternal Antenatal Attachment Scale (MAAS-2). The primary outcome was the STAI-S score at the final visit. A total of 50 participants (25 per group) were required to detect a 20% difference in the primary outcome.

**Results:**

Demographics were comparable between groups. The intervention group demonstrated significantly lower STAI-S scores at mid-pregnancy (46.7 ± 9.3 vs. 52.0 ± 9.0; *p* = 0.023) and at the final visit (43.6 ± 11.8 vs. 51.5 ± 11.5; *p* = 0.004), and higher MAAS-2 scores at the final visit (79.5 ± 6.2 vs. 75.0 ± 6.9; *p* = 0.022). Attachment scores increased significantly during follow-up (4.8 ± 7.3 vs. − 0.36 ± 8.0; *p* = 0.023). Emergency department visits were fewer in the intervention group (3.1 ± 1.5 vs. 4.9 ± 3.3; *p* = 0.024). Regression analyses confirmed independent associations between home ultrasound use, reduced anxiety, and improved attachment.

**Conclusion:**

Telemedicine home ultrasound significantly reduced maternal anxiety and improved antenatal attachment in patients with prior late pregnancy loss. This reassurance strategy may also decrease unscheduled emergency visits, supporting its integration into standard prenatal management in this vulnerable population.

## What does this study add to the clinical work

**Table:** 

In pregnant patients with a prior late pregnancy loss, incorporating telemedicine-guided home ultrasound into routine high-risk care significantly reduces maternal anxiety and enhances antenatal attachment. This reassurance strategy may also decrease unscheduled emergency visits, supporting its integration to standard prenatal management in this vulnerable population.	

## Introduction

Late pregnancy loss is defined as a pregnancy loss beyond 20 weeks of gestation and affects 1:175 patients, with an estimated incidence of 2.6 million patients worldwide annually in the last decades [1, 2]. This experience is associated with maternal life-long psychological morbidity including increased risk for post-traumatic anxiety and major depressive disorders [3, 4].

The subsequent pregnancy following a late pregnancy loss is usually a stressful event, characterized by increased maternal anxiety and decreased antenatal attachment, posing an emotional and psychological burden on the patients and a challenge to the treating medical staff [5, 6]. Moreover, prior studies have demonstrated an association between these traits and adverse neonatal outcomes, especially preterm birth and small-for-gestational-age neonates [7, 8]. A Cochrane review from 2018 delved into recommended care prior to and during subsequent pregnancies following stillbirth and concluded that few evidence-based anxiety-relieving treatments exist, and that further evaluation of psychosocial interventions is an urgent priority [2].

The use of home ultrasound (US) relies on the idea that frequent sonographic fetal assessment may provide assurance for the patients and hopefully reduce anxiety [9]. The recently emerging, telemedicine-guided home US is a feasible, convenient technology, granting good-quality basic scans to assess fetal well-being. The reliability and safety of the device have been previously examined and validated, with an added value of increased patient satisfaction and convenience [10, 11].

Therefore, our aim was to evaluate the effect of incorporating telemedicine home-US sessions into prenatal care on maternal anxiety and antenatal attachment in patients with a history of late pregnancy loss. We hypothesized that the use of this technology in these high-risk patients may contribute by decreasing maternal anxiety and enhancing maternal attachment.

## Materials and methods

In this quasi-randomized trial, we recruited patients with a history of late pregnancy loss during their subsequent pregnancy from a single tertiary center. Eligible patients were recruited from our high-risk pregnancy clinics between 2021 and 2023. Included were patients aged 18–50 years, with a history of pregnancy loss (at or beyond 20 weeks of gestation), and above 20 weeks of their subsequent pregnancy. The exclusion criteria consisted of patients with cellphones incompatible with the home US device, patients with known fetal malformations in the current pregnancy, patients with a diagnosed anxiety disorder and non-Hebrew-speaking patients, as the questionnaires used for outcome assessment in this study were all validated in Hebrew. All patients signed an informed-consent form. This trial received approval from the local ethics committee (number 0126–22-WOMC).

Patients were allocated per day of enrollment and assigned to one of the two study groups, the home US group or the control group. Patients were allocated to the study groups according to the day of enrollment, using a predetermined alternating schedule that assigned participants to either the home ultrasound group or the control group. This allocation approach was chosen to facilitate study logistics in the clinical setting and therefore represents a quasi-randomized design rather than true individual randomization. The patients in the home US group received high-risk pregnancy follow-up according to our departmental protocol with additional twice-weekly telemedicine encounters to assess for basic fetal well-being, including assessment of fetal pulse, movements, and amniotic fluid volume. The US sessions were viewed in real time by a guiding physician who instructed the patients how to operate the device for optimal scans. Notably, the patients could only access the technology with the physicians’ approval in order to prevent misuse and avoid inducing unnecessary anxiety. The control group patients attended our maternal–fetal medicine clinics and received similar high-risk care within our clinics without the additional telemedicine sessions. This included two monthly appointments during which they underwent an ultrasound for fetal weight estimation, biophysical profile, and amniotic fluid volume, as well as a fetal heart rate monitoring (starting at 32 weeks of gestation), and a physician assessment. The ultrasound devices used in this study were provided by the manufacturer, who also offered technical and statistical support. However, the study design, data analysis, interpretation of results, and manuscript preparation were conducted independently by the investigators, without influence from the sponsor.

The outcomes assessed were maternal anxiety and antenatal attachment during the follow-up time. Anxiety was estimated using the widely used State–Trait Anxiety Index (STAI) questionnaire, which includes 20 questions ranked between 20 and 80 points overall, with higher grades indicating higher anxiety. Maternal attachment was assessed using the Maternal Antenatal Attachment Scale (MAAS), which is composed of 19 questions marked between 19 and 95 points in sum, with higher scores reflecting higher maternal attachment.

Participants filled out both questionnaires at three different time points—before initiation of the follow-up period (22–24 weeks), at mid-follow-up (28–30 weeks), and at the end of follow-up (35–37 weeks). The primary outcome examined was maternal anxiety at the end of follow-up, as portrayed by the STAI score on the third questionnaire. The secondary outcomes examined were maternal antenatal attachment### score at the end of follow-up, change in anxiety and attachment scores from first to last questionnaires (calculated as the final score minus the baseline score), pregnancy and neonatal outcomes, including gestational age at delivery, mode of delivery, neonatal birth weight and intensive care unit admission, and emergency department (ED) visits during the follow-up period.

Based on previously published data reporting a mean third-trimester STAI score of approximately 46 among patients with a history of late pregnancy loss [5, 12], we hypothesized that the intervention could reduce anxiety by at least 8 points, which was considered clinically meaningful. Assuming a two-sided alpha level of 0.05 and 80% statistical power to detect this difference between groups, a total sample size of 50 participants (25 per group) was required.

Data were analyzed with SPSS, version 29 (IBM Corp. Released 2021. IBM SPSS Statistics for Windows, Version 29.0. Armonk, NY: IBM Corp). Continuous variables are presented as mean ± standard deviation or median [range] and compared using the Student’s t-test or the non-parametric Mann–Whitney test as appropriate. Categorical variables were calculated as rates (percentages) and compared with Chi-square or Fisher’s exact test as appropriate. All tests were two-tailed, and the threshold for statistical significance was defined as p-value < 0.05. Multivariable linear regression analyses were performed to evaluate the independent association between home ultrasound use and the primary and secondary outcomes, including final anxiety score (STAI-3), final antenatal attachment score (MAAS-3), and number of emergency department visits during follow-up. Covariates included parity, gestational age at the previous pregnancy loss, use of assisted reproductive technology, and use of antidepressant medications. In models evaluating final questionnaire scores, the corresponding baseline score (STAI-1 or MAAS-1) was additionally included to account for baseline differences between participants. Given the exploratory nature of these secondary endpoints, no formal adjustment for multiple comparisons was performed.

## Results

**Fig. 1:**
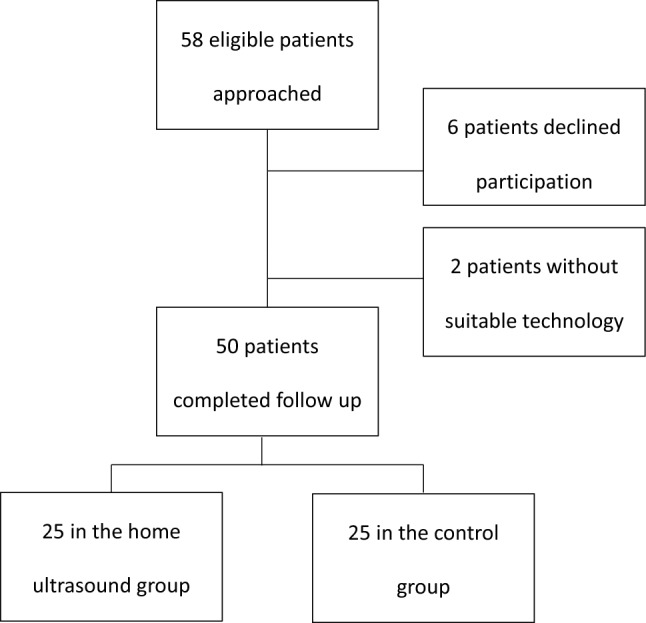
Flowchart describing the study population

During the study period, 58 eligible patients were allocated and approached. Of these, six patients declined participation, and two patients did not have suitable technology for the device. Therefore, a total of 50 patients were recruited and eventually participated in the study, 25 in each group (Fig. 1).

Although not accounted for in the randomization technique, maternal demographics were generally comparable between the two groups, with no significant differences in maternal age, gravidity, parity, gestational age at recruitment, rates of assisted reproductive technology, diabetes, hypertension, and smoking (Table 1).

**Table 1:** Maternal demographics and pregnancy characteristics of the study groups

	Control (*n* = 25)	Home ultrasound (*n* = 25)	*p*-value
Maternal age (years)	31.3 ± 4.7	32.7 ± 4.9	0.345
Gravidity	3.9 ± 1.6	3.6 ± 3.2	0.653
Parity	2.2 ± 1.4	1.6 ± 1.4	0.187
Gestational age at recruitment	25.3 ± 1.9	24.6 ± 0.6	0.466
Assisted reproductive technology	4 (16)	6 (24)	0.725
Any diabetes mellitus	5 (20)	7 (28)	0.741
Any hypertensive disease	2 (8)	1 (4)	1.0
Smoking	3 (12)	3 (12)	1.0
Maternal use of antidepressant therapy	2 (8)	2 (8)	1.0
Week of previous pregnancy loss	28.3 ± 7.1	29.1 ± 6.8	0.734
ED visits during the study period	**4.9 ± 3.3**	**3.1 ± 1.5**	**0.024**

Continuous variables are presented as mean ± SD or median [range] as required and categorical variables as n (%). p-values in bold are statistically significant. *ED* emergency department

Table 2 displays the pregnancy and neonatal outcomes of the study groups. The home US group had significantly fewer emergency department visits during the study period compared to the control group (3.1 ± 1.5 vs. 4.9 ± 3.3 visits, *p* = 0.024). No further significant differences were observed between the groups including gestational age at delivery, mode of delivery, neonatal weight, or neonatal ICU admissions.

**Table 2:** Labor and neonatal outcomes of the study groups

	Control (*n* = 25)	Home ultrasound (*n* = 25)	*p*-value
Gestational age at gestation	37.3 ± 1.1	37.4 ± 1.3	0.840
Vaginal delivery	16 (64)	17 (68)	1.0
Neonatal weight	2904 ± 363	2924 ± 510	0.886
Neonatal ICU admission	4 (16)	7 (28)	0.496

Continuous variables are presented as mean ± SD or median [range] as required and categorical variables as *n* (%). *p*-values in bold are statistically significant. *ICU* intensive care unit

The questionnaire scores are displayed in Table 3. The home US group demonstrated significantly lower anxiety scores at mid-follow-up and at the end of the study period compared to the control group. (46.7 ± 9.3 vs 52.0 ± 9.0, *p* = 0.023 and 43.6 ± 11.8 vs. 51.5 ± 11.5, *p* = 0.004 respectively). Additionally, the home US group had significantly higher maternal antenatal attachment scores at the end of follow-up, as well as a significant increase in attachment score during follow-up time (79.5 ± 6.2 vs. 75.0 ± 6.9, *p* = 0.022 and 4.8 ± 7.3 vs. -0.36 ± 8.0, *p* = 0.023).

**Table 3:** Questionnaire results of the study groups

	Control (*n* = 25)	Home ultrasound (*n* = 25)	*p*-value
STAI-1 score	48.2 ± 14.2	47.1 ± 10.8	0.483
STAI-2 score	**52.0 ± 9.0**	**46.7 ± 9.3**	**0.023**
STAI-3 score	**51.5 ± 11.5**	**43.6 ± 11.8**	**0.004**
∆STAI 3–1	3.6 ± 12.4	− 3.5 ± 14.2	0.091
Number of patients with reduced STAI scores	7 (28)	13 (52)	0.148
MAAS- 1	75.4 ± 8.5	75.1 ± 7.4	0.764
MAAS- 2	76.4 ± 8.5	78.1 ± 7.3	0.509
MAAS- 3	**75.0 ± 6.9**	**79.5 ± 6.2**	**0.022**
∆ MAAS 3–1	**− 0.36 ± 8.0**	**4.8 ± 7.3**	**0.023**
Number of patients with increased MAAS scores	13 (52)	18 (72)	0.243

Continuous variables are presented as mean ± SD and categorical variables as *n* (%). *p*-values in bold are statistically significant. STAI—the State–Trait Anxiety Inventory questionnaire; MAAS—Maternal Antenatal Attachment questionnaire. ∆ scores are calculated as the final score minus the baseline score. All questionnaires designated as questionnaire 1 (STAI /MAAS) were reported between weeks 22–24 of gestation, questionnaire 2 was filled between 28 and 30 weeks and questionnaire 3 was filled between 35 and 37 weeks

Linear regression analyses revealed that lower anxiety scores (STAI-3: *B* = − 7.80, *p* = 0.042) and higher antenatal attachment scores (MAAS-3: *B* = 5.24, *p* = 0.014) were independently associated with the use of the home US device (Table 4).

**Table 4:** Linear regression analyses examining the association between maternal anxiety, antenatal attachment, and emergency department visits to home ultrasound follow-up among patients with previous late pregnancy loss

	Unstandardized B	95% CI for B	*p*-value
STAI-3	– 7.80	[– 15.30, – 0.29]	**0.042**
MAAS-3	5.24	[1.14, 9.36]	**0.014**
Unscheduled ED visits	– 1.46	[– 3.37, 0.45]	0.130

Values reflect the results of multivariate linear regression analyses adjusted for parity, gestational age at previous *IUFD* assisted reproductive technology, use of antidepressants, and baseline STAI or MAAS according to the dependent variable. *STAI* State–Trait Anxiety Index; *MAAS* Maternal Antenatal Attachment Scale; *ED* emergency department

*p*-values in bold are statistically significant

## Discussion

In this quasi-randomized trial, we evaluated the effect of incorporating twice-weekly home US device sessions via telemedicine into prenatal care on maternal anxiety and antenatal attachment in patients with a history of late pregnancy loss. Our findings indicate that integrating home US into prenatal care significantly reduced maternal anxiety, as evident by lower STAI scores at mid and late follow-up. In addition, there was a significant increase in attachment levels during the study duration, as reflected in higher MAAS scores at the end of follow-up and the change in MAAS from baseline to final score. Additionally, the intervention group had fewer unscheduled emergency department visits, suggesting a potential impact on maternal reassurance and healthcare utilization.

A subsequent pregnancy following a late pregnancy loss is an extremely high-risk scenario, both medically and psychologically [1]. The increased anxiety characterizing this pregnancy is a major contributor to maternal distress and is possibly associated with neonatal compromise [7, 8]. Therefore, anxiety-relieving interventions are warranted. While traditional interventions including individual psychological therapy and support groups proved efficient [13, 14], the evidence supporting these studies is deemed weak [15], and limited access and high costs overshadow their feasibility. Effects of different alternative therapies were also examined in association with perinatal anxiety, including mindfulness, yoga practice, biofeedback, physical activity and more, with inconsistent conclusions [16–19]. Our group previously demonstrated that home US reduced anxiety and increased attachment in patients with a history of repeated pregnancy loss [9]. The current study further confirms its feasibility in patients with high-risk pregnancies prone to increased baseline anxiety.

Maternal antenatal attachment following a late pregnancy loss can also be compromised, with data demonstrating disturbed attachment behavior that may even proceed to infancy [20–23]. Moreover, previous studies have demonstrated an association between maternal antenatal anxiety and impaired maternal attachment, emphasizing the need to address the issue of attachment in high-risk pregnancies that may provoke elevated anxiety [24, 25]. Few interventions aimed at increasing antenatal attachment were assessed, including ultrasound, attachment-enhancing programs and psychoeducational interventions. Some of these interventions were deemed successful in increasing antenatal attachment, yet most studies excluded patients with previous late pregnancy loss owing to an expected vulnerable attachment pattern in these patients [26, 27]. This study demonstrates the utility of home US for increasing attachment even in high-risk patients with increased baseline anxiety.

Another pivotal aspect associated with late pregnancy loss is the increased healthcare utilization due to anxiety and insecurity [28]. However, frequent medical encounters might have unlooked consequences, including a financial burden on the patient owing to medical costs and missed work-days, decreased patient satisfaction and elevated costs and burden on the medical system caused by unnecessary excessive medical exams which should all be taken into account [29]. Although portable ultrasound systems require an upfront investment, the reduction in unscheduled emergency department visits observed in our study suggests that this intervention may partially offset these costs by decreasing healthcare utilization. While our study was not designed to formally assess cost-effectiveness, these findings highlight the potential economic benefit of this approach. Future studies should evaluate the cost-effectiveness of telemedicine-guided home ultrasound in high-risk pregnancies.

This study has a few notable strengths including the use of validated anxiety and attachment questionnaires, the fairly long follow-up period and the inclusion of a well-defined high-risk patient population. Additionally, the incorporation of objective outcome measures, such as emergency department visits, strengthens the clinical applicability of our findings.

Notably, our study also has several limitations. First, the relatively small sample size may limit the generalizability of our findings. Second, the exclusion of non-Hebrew-speaking patients may introduce selection bias and limit the applicability of the results to a more diverse population. Third, due to the study design, we are unable to eliminate the stress-relieving effects of the online physician encounters and to isolate the magnitude of the home US effect. However, since this cohort of patients is especially at risk for elevated anxiety in pregnancy, we primarily aimed to ensure no excess anxiety will be caused by participating in this study. Therefore, we decided to provide access to the home US dependent on physician live guidance to prevent excessive use, misuse, or misinterpretation if not handled correctly. Future studies with larger and more diverse populations, along with long-term follow-up, are needed to validate our findings and assess the broader implications of home US technology.

In conclusion, our study confirms that home US reduces maternal anxiety and increases antenatal attachment, by offering frequent fetal reassurance in a controlled and supervised manner, in patients with a history of late pregnancy loss. Moreover, this intervention may decrease visits to unscheduled medical follow-up, thereby decreasing medical costs. These findings highlight the potential of telemedicine-guided fetal assessment as a valuable adjunct in the care of high-risk pregnancies. Further research is warranted to optimize implementation strategies and assess the long-term impact on both maternal and neonatal outcomes.

## Data Availability

All data are available upon reasonable request from the corresponding author.
